# Episodic and Semantic Autobiographical Memory in Mild Cognitive Impairment (MCI): A Systematic Review

**DOI:** 10.3390/jcm12082856

**Published:** 2023-04-13

**Authors:** Giulia Marselli, Francesca Favieri, Maria Casagrande

**Affiliations:** 1 Department of Psychology, “Sapienza” University of Rome, 00185 Rome, Italy; 2 Department of Dynamic and Clinical Psychology and Health Studies, “Sapienza” University of Rome, 00185 Rome, Italy; francesca.favieri@uniroma1.it

**Keywords:** autobiographical memory, episodic memory, semantic memory, mild cognitive impairment, MCI

## Abstract

Introduction: Mild cognitive impairment (MCI) is a syndrome defined as a decline in cognitive performance greater than expected for an individual according to age and education level, not interfering notably with daily life activities. Many studies have focused on the memory domain in the analysis of MCI and more severe cases of dementia. One specific memory system is represented by autobiographical memory (AM), which has been largely studied in Alzheimer’s disease and its effect on AM; however, the impairment of AM in moderate forms of decline, such as MCI, is still controversial. Objective: The main aim of this systematic review is to analyze the functioning of autobiographical memory in patients with MCI, considering both the semantic and the episodic components. Materials: The review process was conducted according to the PRISMA statement. The search was conducted until 20 February 2023 in the following bibliographical databases: PubMed, Web of Science, Scopus, and PsycInfo, and twenty-one articles were included. Results: The results highlight controversial findings concerning the semantic component of AM since only seven studies have found a worse semantic AM performance in patients with MCI compared to the HC group. The results of impaired episodic AM in individuals with MCI are more consistent than those concerning semantic AM. Conclusions: Starting from the evidence of this systematic review, further studies should detect and investigate the cognitive and emotional mechanisms that undermine AM performance, allowing the development of specific interventions targeting these mechanisms.

## 1. Introduction

The term mild cognitive impairment (MCI) indicates a syndrome conceptualized as a decline in cognition more severe than expected for an individual according to age and schooling. This impairment does not notably interfere with daily activities [1]. Since its first proposal [2], the MCI construct has been evolving: it was originally used to describe a pattern of impairments involving the memory domain, while afterward, it broadened to embrace many other domains [3]. Petersen [4] hypothesized a categorization into multiple subtypes. This hypothesis distinguishes MCI into (a) amnestic MCI single domain, in which there is an impairment in the memory domain only (aMCIsd); (b) amnestic MCI multiple domains, characterized by impairments in memory and other cognitive domains (aMCImd); (c) non-amnestic MCI single domain, marked by an impairment in one domain that is not memory (naMCIsd); and, finally, (d) non-amnestic MCI multiple domains, characterized by at least two impairments in two cognitive domains other than memory (naMCImd). These subtypes have different development pathways [5], and individuals with aMCI convert more frequently into Alzheimer’s disease (AD) [5]. Therefore, many studies have focused on the memory domain since it remains relevant in the analysis and outcome of MCI, as in more severe cases of dementia.

One specific memory system is represented by autobiographical memory (AM), which is important for social functioning [6] since it gives people the sense of a subjective timeline. Thanks to AM, individuals can mentally travel back, acquiring a sense of “self” that can exist in the subjective time [7]. AM goes beyond the mere recall of past events: it creates a sense of extended self through time in order to reflect and evaluate events related to the self [8]. Therefore, AM represents a special form of memory that regards the individual’s life experiences, and it comprises multiple forms of long-term memory [9], including episodic and semantic components [10]. Autobiographical episodic memory refers to the recall of specific episodes from one’s past, such as an unexpected accident. On the other hand, autobiographical semantic memory refers to the general knowledge about the self, such as the name of the street in which one lived as a child. Investigating the functioning of autobiographical memory and its different phenomenological aspects in both physiological and pathological decline occurring with aging is a challenge of the current study [11], relevant for its role in integrating, interpreting, and evaluating past events and self [12].

In healthy aging, it has been demonstrated that AM declines with aging and that the episodic component is more affected than the semantic one [11]. Piolino et al. [11] found that episodic AM decline becomes apparent after 60 years. In pathological aging, while the impact of Alzheimer’s disease on AM is largely studied [13,14], it is still controversial in moderate forms of decline, such as MCI. Recent studies have evidenced episodic AM impairments in patients with MCI [15,16]. While recalling past events, these studies found that patients with aMCI had a worse performance in episodic memory but recalled more semantic details. Patients with aMCI typically show a malfunctioning of the hippocampus [17], which could cause an isolated impairment of episodic memory [15]. However, there is still a lack of clear evidence about the effect of MCI on AM and its components.

Therefore, the main objective of this systematic review is to analyze the functioning of autobiographical memory in patients with MCI, considering both the semantic and the episodic components.

## 2. Method

### 2.1. Research Questions

This systematic review aims to answer the following research questions:

1. Is autobiographical memory impaired, and to what degree in patients with MCI?
2. Which components of AM are impaired and how are they impaired in MCI?

### 2.2. Search Strategy

The present systematic review adhered to the guidelines developed by the PRISMA-Statement [18,19] and was registered on the Open Science Framework (https://osf.io/dn25x, accessed on 10 November 2022). The search was conducted until 20 February 2023 in the following bibliographical databases: PubMed, Web of Science, Scopus, and PsycInfo. The review is based only on English, Italian, French, and Spanish articles. The search syntax can be found in Table 1.

### 2.3. Inclusion/Exclusion Criteria

According to the objectives of this review, the following inclusion criteria have been adopted: (I) randomized cross-sectional or longitudinal studies that aim at evaluating autobiographical memory in patients with MCI; (II) articles that specify the instruments for evaluating autobiographical memory; (III) studies that include a healthy control group; (IV) studies that clearly specify which criteria have been used for diagnosing MCI; (V) studies that analyzed differences in autobiographical memory performance between the different subtypes of MCI.

Specific exclusion criteria were: (I) studies that included clinical patients (people with neurological conditions, with metabolic or autoimmune diseases, with cardiovascular or oncological problems, or with diagnoses of dementia); (II) studies that included other diagnoses of cognitive impairment such as CIND, AAMI, or AACD; (III) studies that measured autobiographical memory only with the use of neurophysiological measures; (IV) gray literature (pre-print papers that have not undergone the peer-review process, Ph.D. dissertations).

**Figure 1:**
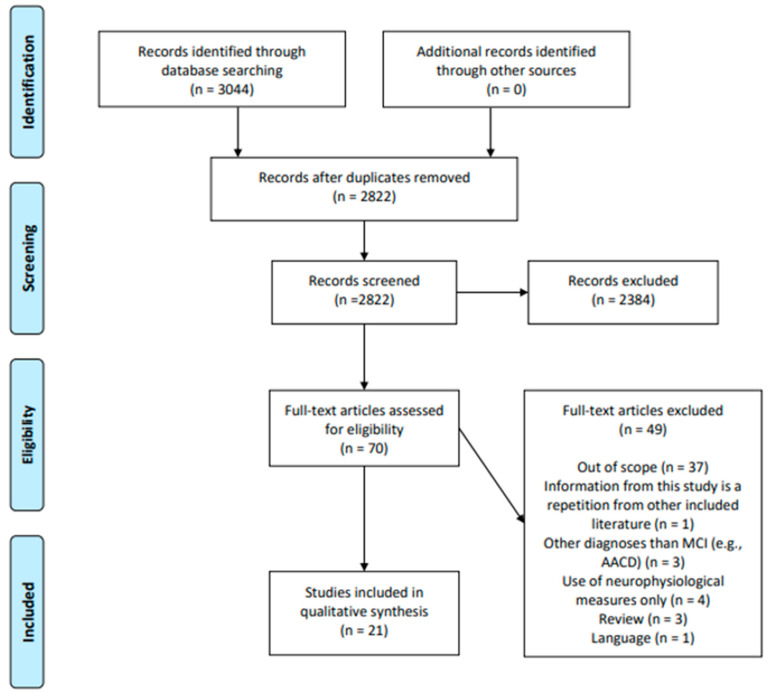
PRISMA flow diagram.

Two researchers independently performed a screening of the selected articles. By screening titles and abstracts, non-relevant articles were excluded, which allowed including 70 studies. Afterward, the reading of the full texts resulted in 21 retained articles. This process is described in Figure 1.

### 2.4. Studies’ Risk of Bias

The present systematic review adopted the Cochrane Collaboration’s tool for assessing the risk of bias [20]. For this systematic review, we considered the following risk of biases:

(i) Attrition bias (which can be defined as the bias consequent of the presence of incomplete outcome data);
(ii) Reporting bias (i.e., the bias resulting from the reporting of selective outcomes or the absence of reporting relevant results; reporting bias was considered low risk if all prespecified outcomes were reported, as suggested by Higgins et al. [20]).

As a complement to these types of biases, two other biases were included:

(i) Sample bias (i.e., bias resulting in samples that do not represent the general population, undermining the generalization of results, or lack of demographic information, such as female ratio, mean age, or mean schooling years);
(ii) Measurement bias (i.e., bias due to using non-validated tasks to measure autobiographical memory).

A summary of the risk of bias in reviewed studies can be found in Table 2. Nine studies [16,21,22,23,24,25,26,27,28] did not meet any of the considered bias. Attrition and reporting biases posed low risks in all of the included studies. Instead, sample bias risk was high in six articles [15,29,30,31,32,33], due to the lack of demographic information (e.g., female ratio, mean age, mean years of schooling). Moreover, measurement bias risk was high in eight studies [4,5,6,7,8,9,10,11,12,13,14,15,16,17,18,19,20,21,22,23,24,25,26,27,28,29,30,31,32,33,34,35,36,37,38,39] due to the use of non-validated or ad hoc tasks to measure autobiographical memory.

## 3. Results

### 3.1. Overview

The twenty-one articles included in this systematic review involved 1046 participants. Among these, 462 were included in the MCI group, while 584 were included in the healthy control group. The female percentage ranged between 23% and 79% in the MCI group and between 44% and 86% in the HC group. The mean age ranged between 63.13 years (SD = 5.78) and 81.8 (SD = 7.8) years in the MCI group and between 62.94 (SD = 5.73) years and 78.35 (SD = 5.75) years in the healthy controls. The mean years of schooling varied from 7.6 (SD = 2.6) to 16.68 (SD = 3.96) in the MCI group and from 7.9 (SD = 2.5) to 16.06 (SD = 2.80). Five articles did not report years of education [29,30,31,32,33]. Moreover, all the studies have been conducted in Europe, except for six articles that have been placed either in Canada [16,21,27,36] or in Australia [29,30]. These characteristics are summarized in Table 3.

### 3.2. MCI Diagnosis

In this systematic review, 20 studies out of 21 based their diagnosis of MCI on Petersen’s criteria. Only the study of Irish et al. [23] referred to Winblad’s criteria. These criteria are more thoroughly described in Table 4.

Twenty studies out of twenty-one focused on one specific subtype of MCI, that is, the amnestic subtype. Since the studies included in this systematic review intended to focus on autobiographical memory impairments, most articles have not considered the non-amnestic MCI subtype. Only the study by Davidson et al. [36] has not specified whether they included patients with MCI in general or if they focused only on the amnestic subtype.

In Table 5, it is possible to observe the assessed cognitive domains and the neuropsychological tests that the authors have utilized in their studies.

### 3.3. Episodic and Semantic Autobiographical Memory

All the studies included in this systematic review report an impairment in the episodic component of AM in patients with MCI compared to healthy controls.

The situation is more controversial for what concerns the semantic component. Fourteen studies out of twenty-one have studied this aspect, while seven articles [26,32,34,35,36,37,38] focused only on the episodic component. Among the 14 articles that analyzed autobiographical semantic memory, 4 of them [21,24,29,33] have not found any significant differences in semantic memory scores between patients with MCI and healthy controls. On the other hand, seven studies [22,23,25,28,30,31,39] have found a worse semantic performance in patients with MCI when compared to the HC group. The other three studies [15,16,27] found more semantic details in patients’ recollections than controls.

### 3.4. Internal and External Details

In five out of twenty-one studies [15,16,21,27,34], autobiographical memory was assessed following the protocol that was developed and standardized by Levine et al. [40]. According to this procedure, the autobiographical memories were transcribed and segmented in order to distinguish external and internal details. Internal details corresponded to episodic memory since they reflected information regarding the main event. On the other hand, external details were not specific to the main episode and were scored as semantic memory (concerning general knowledge of facts or events related to the self).

Three [15,16,27] out of the five articles that used this method found that controls produced more internal details (episodic memories) than patients with aMCI, whereas patients with aMCI produced more external details (semantic memories) than controls. Therefore, patients with aMCI recall fewer episodic, event-specific details and more semantic details. The remaining two studies [21,34] observed the same trend for internal details: patients with MCI recalled fewer internal details than controls. However, they did not detect a significant difference in the amount of recalled external details.

### 3.5. Temporal Gradient

Regarding the recall of autobiographical memories, some authors [41] found evidence of a significant Ribot-like temporal gradient in patients’ performance, with better preservation of remote memories than recent ones.

In our review, 13 studies [16,21,22,23,24,25,26,28,31,34,35,37,39] examined the temporal gradient in the recall of episodic autobiographical memories in patients with MCI. Three articles [21,22,23] did not find significant differences in time-period performance. Seven studies [24,25,26,28,31,37,39] confirmed Ribot’s law, showing that, in patients with MCI, recent episodic memories are more likely to be lost than the more remote memories. However, three articles [16,34,35] found the opposite effect: they detected better scores for recent events than more remote ones.

Regarding semantic memory, nine studies [16,21,22,23,24,25,28,31,39] analyzed the recall temporal gradient for patients with MCI. Results are more controversial when compared to episodic memory. Two articles [22,23] have not found significant differences in the time-period performance. On the other hand, only three studies [24,25,31] confirmed Ribot’s law for semantic memory, while four articles [16,21,28,39] found the opposite effect, with recent semantic memories being better preserved than remote ones. These results are summarized in Table 6.

**Table 3:** Selected studies’ characteristics.

Authors	Country	N	Groups	Sex (% F)	Mean Age (SD)	Mean Schooling Years (SD)	MCI Diagnostic Criteria	AM Assessment	Episodic/ Semantic	Cognitive Assessment	Results
Barnabe et al., 2012 [21]	Canada	20 20	aMCI HC	40% 70%	76.40 (6.87) 78.35 (5.75)	14.60 (4.30) 14.45 (2.74)	Petersen et al., 2001 [5]	AMI [42]; Slightly modified version of the AI [40].	Episodic and semantic	MoCA; MMSE; LM-II WMS-III; Full battery of standardized neuropsychological measures.	Using AMI, HC had a better performance than patients with MCI (*p* < 0.01) in autobiographical episodic memory, and there were no differences in autobiographical semantic memory (*p* = 0.16). Using AI, HC recalled more internal details than the MCI (*p* < 0.001) group, while there were no differences in external details.
Bastin et al., 2013 [34]	Belgium	35 24	aMCI HC	34% 75%	73.9 (6.6) 73.2 (7.2)	13 (3.5) 12.5 (2.8)	Petersen and Negash, 2008 [43]	Episodic Autobiographical Memory Questionnaire	Episodic	Mill Hill vocabulary; Episodic memory cued recall; Episodic memory recognition (remember/know/guess); Episodic memory continuous recognition; Reading span; Semantic memory cued recall; Semantic memory recognition; Hayling test.	HC recalled more internal details than aMCI (post hoc Tukey tests, *p* < 0.05), while there were no differences in external details (*p* > 0.71).
Berna et al., 2012 [35]	Germany	63 138	MCI HC	46% 53%	74.02 (0.87) 73.84 (0.89)	12.29 (2.11) 13.84 (3.04)	Petersen et al., 2001 [5]	Semi-structured interview that assesses the episodic component.	Episodic	NAI; Aufmerksamkeits-Belastungs test; Similarities subtest of the HAWIE-R; Verbal fluency subtest from the Leistungsprufsystem; Raumliche Vorstellung from the Leistungsprüfsystem	HC had a better performance in autobiographical episodic memory than MCI (*p* = 0.02).
Bizzozero et al., 2012 [22]	Italy	19 19	aMCI HC	79% 79%	74.9 (4.7) 75 (4.4)	7.6 (2.6) 7.9 (2.5)	Petersen et al., 1999 [2]	AM enquiry by Borrini et al. [44]	Episodic and semantic	CDR; MODA	HC had a better performance than aMCI (t = 4.33, df = 36, *p* < 0.0001) in the overall autobiographical memory. After distinguishing a posteriori the contribution of the “personal semantics” component and the episodic component, it was shown that HC performed better in both of them compared to aMCI.
Buckley et al., 2014 [29]	Australia	11 31	MCI HC	46% 48%	79.09 (7.3) 77.23 (7.2)	-	Petersen et al., 1999 [2]	EAMI [45]	Episodic and semantic	CVLT-II short delay free recall and long delay free recall; LM WMS immediate and delayed recall measures (Story 1 only); RCFT 30 min delayed recall; CANTABeclipse v3.0 PAL Stage 6	HC (M = 0.01, SD = 1.0) had a better performance in episodic autobiographical memory than participants with MCI (M = −1.00, SD = 0.9). Instead, there were no significant differences in autobiographical semantic memory between the MCI group (M = −0.60, SD = 1.1) and the HC group (M = 0.03, SD = 0.8).
Buckley et al., 2014 [30]	Australia	43 43	MCI HC	58% 56%	79.6 (6.9) 73.77 (6.1)	-	Winblad et al., 2004 [46]	EAMI [45]	Episodic and semantic	CVLT-II new learning, post-interference recall, delayed recall, and recognition measures; LM WMS immediate and delayed recall measures; RCFT 30-min delayed recall and recognition; FFS; Stroop test; 30-item BNT	MCI participants performed significantly worse on episodic autobiographical memory recall (M = 3.53, SD = 2) than HC (M = 5.16, SD = 1.2). Moreover, they also performed significantly worse on autobiographical semantic memory (M = 9.70, SD = 4.2) than the HC group (M = 12.91, SD = 1.3).
Davidson et al., 2016 [36]	Canada	19 34	MCI HC	53% 62%	75.63 (6.23) 70.09 (4.32)	16.68 (3.96) 16.06 (2.80)	Petersen et al., 1999 [2]	Ad hoc telephone questionnaire regarding the lab visit (from 1 to 13 days after the visit).	Episodic	MoCA; WCST; Forward and reverse DS from WAIS-III; Stroop test; BNT; 1 min letter (F, A, and S) and category (animal) fluency; LM—I and LM—II from WMS; CVLT-II; 5-word delayed recall subtest from the MoCA.	MCI had a worse performance than HC in remembering the details of the episodic event.
De Simone et al., 2017 [37]	Italy	18 18	aMCI HC	55% 55%	73.4 (6.3) 71.4 (7.8)	12.1 (3.7) 13.7 (3.2)	Petersen et al., 2014 [47]	Ad hoc measure in which participants were asked to recall the personal events that occurred when they first learned about 50 famous events that were previously selected.	Episodic	MMSE	HC group had a better performance (mean 2 SD ± 0.88) than the aMCI group (mean 1.07 SD ± 1.06).
Donix et al., 2010 [38]	Germany	16 16	aMCI HC	44% 56%	63.13 (5.78) 62.94 (5.73)	9 (4.3) 9.82 (4.22)	Petersen, 2004 [48]	ABM task [49]	Episodic	MMSE; CVLT	HC had fewer extended (*p* = 0.004) memories and an increased number of specific memories (*p* < 0.001). Therefore, participants with aMCI showed less specificity than HC in episodic autobiographical memory.
Gamboz et al., 2010 [15]	Italy	14 14	aMCI HC	-	74.7 (7.4) 73.5 (8)	12.8 (5.1) 13 (2)	Petersen et al., 1999 [2]	Subjects had to respond to eight cue words, recalling (or imagining) four episodes (that occurred or will occur in the past or next year within their life)	Episodic and semantic	MMSE; MDB; FAB	HC produced more internal details (M = 7.42; SD = 1.98) than aMCI (M = 4.42; SD = 1.87), t (27) = 4.11, *p* < 0.0001. aMCI produced more external details (M = 6.31; SD = 2.26) than HC (M = 3.23; SD = 1.36), t (27) =−4.37, *p* < 0.0001.
Irish et al., 2010	Ireland	16 18	aMCI HC	37% 78%	71.8 (6.8) 76 (4.3)	13.8 (4.7) 14 (3.1)	Winblad et al., 2004 [46]	EAMI [45]	Episodic and semantic	MMSE; CDT; NART; Digit and spatial span (WMS-III); Letter and category fluency; TMT; Stroop test.	HC had a better performance than aMCI in autobiographical semantic memory (F (1,32 = 27.963; *p* < 0.0001) across all periods except childhood (*p* = 0.627) and early adulthood (*p* = 0.066). HC had a better performance than aMCI in episodic autobiographical memory (F (1,32) = 69.211; *p* < 0.0001) across all periods.
Leyhe et al., 2009 [24]	Germany	20 20	aMCI HC	40% 70%	72.6 (6.8) 71.6 (6.5)	10.2 (5.2) 11.8 (2.8)	Petersen et al., 1999 [2]	AMI [42]	Episodic and semantic	CERAD	HC had a better performance than aMCI (*p* < 0.05) in episodic autobiographical memory, while there were no differences in autobiographical semantic memory (*p* = 0.072).
Meléndez et al., 2016 [31]	Spain	15 29	aMCI HC	73% 86%	81.8 (7.8) 78.2 (5.1)	-	Petersen et al., 2001 [5]	AMI [42]	Episodic and semantic	MMSE	HC had a better performance than aMCI in episodic autobiographical memory across all periods (*p* < 0.001). HC had a better performance than aMCI in autobiographical semantic memory, only in the recent life stage (*p* < 0.001).
Meléndez et al., 2019 [32]	Spain	32 32	aMCI HC	62% 56%	76.50 (5.44) 74.21 (4.67)	-	Petersen, 2004 [48]	AMT [49]	Episodic	GDS; MMSE; Categorical and phonological verbal fluency from the TBR; TAVEC-I; TAVED-D; DSB and DSF of the WAIS-III; Copy and reproduction of complex geometric figures from Rey’s memory test	HC had an increased number of specific responses than MCI (*p* = 0.010).
Meléndez et al., 2021 [33]	Spain	17 26	aMCI HC	65% 61%	77.35 (4.76) 74.53 (4.90)	-	Petersen, 2004 [48]	AMI [42]	Episodic and semantic	MMSE; VFTC; VFTP; TAVEC-I; TAVEC-D; DSF; DSB; Rey-I; Rey-D	HC had a better performance than aMCI in episodic autobiographical memory (*p* = 0.010), while there were no differences in autobiographical semantic memory.
Müller et al., 2013 [25]	Germany	20 20	aMCI HC	60% 65%	72.6 (6.8) 71.9 (6.5)	13.2 (5.2) 13.1 (2.6)	Petersen et al., 1999 [2]	AMI [42]	Episodic and semantic	MMSE; TMT part B; CERAD word list immediate and delayed recall; CERAD word list recognition; Verbal learning of 10 words over 3 trials, as well as recall and recognition of the 10-word list	aMCI has a significant different performance (*p* < 0.01) in the autobiographical episodic memory for recent life experiences compared to HC. Moreover, aMCI has a significantly different performance (*p* < 0.05) in the autobiographical semantic memory for recent life experiences compared to HC.
Müller et al., 2016 [26]	Germany	20 21	aMCI HC	45% 48%	73 (4.5) 72.4 (6.5)	11.6 (3.4) 12.2 (3.2)	Petersen et al., 1999 [2]	AMI [42]	Episodic	MMSE; A 15-item short version of the BNT; Semantic word fluency test (animals, 1 min); Word list learning (10 words, 3 trials); Word list recall after distraction; Word list recognition (10 target and 10 distractor words); Figure copying; Delayed figure recall	HC had a better performance than aMCI in episodic autobiographical memories from early adulthood (*p* = 0.04) and recent life (*p* < 0.001), while there were no significant differences for the childhood period (*p* = 0.06).
Murphy et al., 2008 [16]	Canada	17 18	aMCI HC	59% 44%	76.2 (5.7) 74.2 (6.4)	14.5 (2.8) 13.6 (3.5)	Petersen, 2004 [48]	AI [40]	Episodic and semantic	HVLT-R; BVMT-R; LM or verbal paired associates; DS; BNT; RCFT copy; Trail-making Test	HC recalled an increased number of internal details (M = 89.83; SD = 39.21) than aMCI (M = 63.18; SD = 22.12). aMCI recalled more external details (M = 98.12; SD = 54.92) than HC (M = 62.39; SD = 27.42).
Serra et al., 2020 [39]	Italy	17 13	aMCI HC	23% 61%	71.8 (6.2) 69.6 (5.9)	12.2 (4.2) 14.1 (2.7)	Albert et al., 2011 [1]	Modified version [50] of the AMI [42]	Episodic and semantic	Immediate and 15 min delayed recall of a 15-word list test; Immediate and 20 min delayed recall of a short story test; Immediate and 20 min delayed recall of the RCFT; DS; Corsi block tapping task forward and backward; Phonological word fluency; Modified card-sorting test; Naming objects subtest of the battery for the analysis of aphasic deficits; Raven’s colored progressive matrices; Copy of simple drawings; Copy of drawings with landmarks; Copy of RCFT	HC performed better than aMCI in both the episodic and semantic autobiographical memory components.
Sheldon et al., 2015 [27]	Canada	16 16	aMCI HC	38% 69%	75.1 (5.7) 74.4 (7.4)	15 (2.9) 15.1 (3)	Petersen, 2004 [48]	AI [40]	Episodic and semantic	NART; MMSE; TMT part B; Color-word Stroop test; RCFT Copy; HVLT-R; WMS-R LM (immediate and delay recall); RCFT immediate recall.	HC produced more internal details (*p* = 0.09, d = 0.61) and fewer external details (*p* < 0.05, d = 0.79) than aMCI.
Tramoni et al., 2012 [28]	France	14 14	aMCI HC	57% 57%	75.1 (6.4) 70.4 (8.7)	9.92 (3.43) 9.64 (2.59)	Petersen et al., 2001 [5]	AMI [42]; Test of familiar photographs	Episodic and semantic	MMSE; RL/RI-16; DMS48; WMS-III LM; WAIS-III information subtest; Picture-naming task; WAIS-III matrix reasoning subtest; TMT; Word fluency letter (P); Word fluency category (animal); WAIS-III digit span subtest; Benton face perception; Benton line orientation	AMI: HC had a better performance than aMCI in both the episodic and the semantic autobiographical memory components, despite the time epochs. Test of familiar photographs: HC had a better performance than aMCI only for recently experienced episodes

**Table 4:** Diagnostic criteria.

Author	Diagnostic Criteria	Global Functioning	Subjective Complaint of Cognitive Decline	Objective Cognitive Impairment	Intact Functional Abilities	Absence of Dementia	Normal Mental Status
Barnabe et al., 2012 [21]	Petersen et al., 2001 [5]		√	<1 SD	√	√	√
Bastin et al., 2013 [34]	Petersen and Negash, 2008 [43]			√	√	√	
Berna et al., 2012 [35]	Petersen et al., 2001 [5]		√	<1 SD	√	√	√
Bizzozero et al., 2012 [22]	Petersen et al., 1999 [2]	CDR = 0.5		<5% of the inferential tolerance limits in at least one task assessing the memory domain			
Buckley et al., 2014 [29]	Petersen et al., 1999 [2]		√	<1.5 SD	√		
Buckley et al., 2014 [30]	Winblad et al., 2004 [46]		√	<1.5 SD in tasks assessing the memory domain	√		
Davidson et al., 2016 [36]	Petersen et al., 1999 [2]	-	-	-	-	-	-
De Simone et al., 2017 [37]	Petersen et al., 2014 [47]	MMSE > 23.8	√	Scoring below age/education adjusted norms on at least one task assessing the memory domain		√	
Donix et al., 2010 [38]	Petersen, 2004 [48]	-	-	<1 SD	-	-	-
Gamboz et al., 2010 [15]	Petersen et al., 1999 [2]	MMSE ≥ 26	√	<1.5 SD in tasks assessing the memory domain	√	√	√
Irish et al., 2010 [23]	Winblad et al., 2004 [46]		√	<1.5 SD	√	√	
Leyhe et al., 2009 [24]	Petersen et al.,1999 [2]		√	<1 SD in the delayed word recall	√	√	√
Meléndez et al., 2016 [31]	Petersen et al., 2001 [5]		√	MMSE < 23	√	√	√
Meléndez et al., 2019 [32]	Petersen, 2004 [48]	-	-	-	-	-	-
Meléndez et al., 2021 [33]	Petersen, 2004 [48]	At levels 2 and 3 on the GDS					
Müller et al., 2013 [25]	Petersen et al., 1999 [2]		√	√	√	√	√
Müller et al., 2016 [26]	Petersen et al., 1999 [2]		√	√	√	√	√
Murphy et al., 2008 [16]	Petersen, 2004 [48]	Scores within 1 SD of the mean based on normative age data on the following tasks: MMSE, digit span, Boston naming test, Rey–Osterrieth complex figure copy and trail-making test	√	Scoring below age/education/IQ adjusted norms in at least two tasks assessing memory	√	√	√
Serra et al., 2020 [39]	Albert et al., 2011 [1]	MMSE > 23.8	√	Scoring below age/education adjusted norms on at least one task assessing the memory domain	√		
Sheldon et al., 2015 [27]	Petersen, 2004 [48]		√	“Typical” < 1.5 SD in at least one task or “comprehensive” < 1 SD in at least two tasks	√		√
Tramoni et al., 2012 [28]	Petersen et al., 2001 [5]	MMSE > percentile 10	√	<1.5 SD in RL/RI-16	CDR = 0.5 IADL = 0	√	

**Table 5:** Assessed cognitive domains and neuropsychological tests for MCI diagnosis.

Author	Diagnostic Criteria	Assessed Cognitive Domains								
		Global Functioning	Intelligence	Memory	Attention	Executive Functions	Language	Praxia	Visuospatial Ability	Processing Speed
Barnabe et al., 2012 [21]	Petersen et al., 2001 [5]	MMSE; MoCA		LM-II WSM-III						
Bastin et al., 2013 [34]	Petersen and Negash, 2008 [43]		Mill Hill vocabulary	Episodic memory cued recall; Episodic memory recognition (remember/know/guess); Episodic memory continuous recognition; Semantic memory cued recall; Semantic memory recognition		Reading span; Hayling test				
Berna et al., 2012 [35]	Petersen et al., 2001 [5]		Similarities subtest of the HAWIE-R	NAI	Aufmerksamkeits-Belastungs-Test.		Verbal fluency subtest from the Leistungsprufsystem.		Raumliche Vorstellung from the Leistungsprüfsystem.	
Bizzozero et al., 2012 [22]	Petersen et al., 1999 [2]	CDR		MODA (prose memory, paired associates, and supraspan non-verbal learning)						
Buckley et al., 2014 [29]	Petersen et al., 1999 [2]			CVLT-II short delay free recall and long delay free recall; WMS LM immediate and delayed recall measures (Story 1 only); RCFT 30 min delayed recall; CANTABeclipse v3.0 PAL Stage 6						
Buckley et al., 2014 [30]	Winblad et al., 2004 [46]			CVLT-II new learning, post-interference recall, delayed recall, and measure; WMS LM immediate and delayed recall measure; RCFT 30 min delayed recall and recognition		FFS; Stroop test	30-item BNT			
Davidson et al., 2016 [36]	Petersen et al., 1999 [2]	MoCA		LM—I and LM—II from WMS; CVLT-II; 5-word delayed recall subtest from the MoCA		WCST; Forward and reverse DS from WAIS III; Stroop test	BNT; 1 min letter (F, A, and S) and category (animal) fluency			
De Simone et al., 2017 [37]	Petersen et al., 2014 [47]	MMSE								
Donix et al., 2010 [38]	Petersen, 2004 [48]	MMSE		CVLT						
Gamboz et al., 2010 [15]	Petersen et al., 1999 [2]	MMSE		Episodic memory tasks included in the MDB						
Irish et al., 2010 [23]	Winblad et al., 2004 [46]	MMSE	NART			Digit and spatial span (WMS-III); letter and category fluency; TMT; Stroop test			CDT	
Leyhe et al., 2009 [24]	Petersen et al., 1999 [2]	MMSE		Delayed word recall (CERAD)						
Meléndez et al., 2016 [31]	Petersen et al., 2001 [5]	MMSE								
Meléndez et al., 2019 [32]	Petersen, 2004 [48]	-	-	-	-	-	-	-	-	-
Meléndez et al., 2021 [33]	Petersen, 2004 [48]	MMSE	DSF; DSB	TAVEC-I; TAVEC-D; Rey-D			VFTC; VFTP		Rey-I	
Müller et al., 2013 [25]	Petersen et al., 1999 [2]	MMSE		CERAD word list immediate and delayed recall; CERAD word list recognition; verbal learning of 10 words over 3 trials, as well as recall and recognition of the 10-word list						
Müller et al., 2016 [26]	Petersen et al., 1999 [2]	MMSE		Word list learning (10 words, 3 trials); word list recall after distraction; word list recognition (10 target and 10 distractor words); delayed figure recall			A 15-item short version of the BNT; semantic word fluency test (animals, 1 min)			
Murphy et al., 2008 [16]	Petersen, 2004 [48]	MMSE		HVLT-R; BVMT-R; LM or verbal paired associates						
Serra et al., 2020 [39]	Albert et al., 2011 [1]		Raven’s colored progressive matrices;	Immediate and 15 min Delayed recall of a 15-word list test; immediate and 20 min delayed recall of a short story test; immediate and 20 min delayed recall of the RCFT; DS; Corsi block-tapping task forward and backward		Phonological word fluency; modified card sorting test	Naming objects subtest of the battery for the analysis of aphasic deficits	Copy of simple drawings; copy of drawings with landmarks; copy of RCFT		
Sheldon et al., 2015 [27]	Petersen, 2004 [48]	MMSE		HVLT-R; WMS-R LM subtest (immediate and delay recall); RCFT immediate recall						
Tramoni et al., 2012 [28]	Petersen et al., 2001 [5]	MMSE		RL/RI-16; DMS48; WMS-III LM		WAIS-III matrix reasoning subtest; TMT; word fluency letter (P); word fluency category (animal); WAIS-III DS	WAIS-III information subtest; picture-naming task		Benton face perception; Benton line orientation	

**Table 6:** Temporal gradient in the autobiographical memory performance in MCI patients.

Author	Episodic Memory	Semantic Memory
Barnabe et al., 2012 [21]	n.s.	Recent > childhood
Bastin et al., 2013 [34]	Recent > remote	-
Berna et al., 2012 [35]	Recent > school period early adulthood > school period	-
Bizzozero et al., 2012 [22]	n.s.	n.s.
Buckley et al., 2014 [29]	-	-
Buckley et al., 2014 [30]	-	-
Davidson et al., 2016 [36]	-	-
De Simone et al., 2017 [37]	Remote > recent	-
Donix et al., 2010 [38]	-	-
Gamboz et al., 2010 [15]	-	-
Irish et al., 2010 [23]	n.s.	n.s.
Leyhe et al., 2009 [24]	Childhood > recent early adulthood > recent	Childhood > recent early adulthood > recent
Meléndez et al., 2016 [31]	Childhood > recent early adulthood > recent	Early adulthood > childhood early adulthood > recent
Meléndez et al., 2019 [32]	-	-
Meléndez et al., 2021 [33]	-	-
Müller et al., 2013 [25]	Childhood > recent	Childhood > recent early adulthood > recent
Müller et al., 2016 [26]	Childhood > early adulthood childhood > recent early adulthood > recent	-
Murphy et al., 2008 [16]	Recent > remote	Recent > remote
Serra et al., 2020 [39]	Remote > recent	Recent > remote
Sheldon et al., 2015 [27]	-	-
Tramoni et al., 2012 [28]	Childhood > recent	Recent > childhood

## 4. Discussion

This systematic review aimed to analyze AM performance in patients with MCI, specifically focusing on the main features of AM alteration. Generally, the large number of studies included in the first screening can confirm the interest in this topic. AM has a critical role in forming one’s identity, and impairments in AM can have devastating consequences for patients and their families [51]. These aspects have led us to focus on this specific memory component to study the level of impairment that affects patients with MCI. Moreover, we aimed to understand further whether the episodic (i.e., the recall of specific episodes from one’s past) or the semantic (i.e., the general knowledge about the self) components of AM are impaired in patients with MCI. This systematic review highlights the presence of impairments in episodic autobiographical memory. However, results are more controversial when assessing the semantic component of AM.

Firstly, it is important to underline that these results are only generalizable to some MCI subjects since 20 out of 21 studies focused on 1 specific subtype of MCI, that is, the amnestic subtype. It is known that individuals affected by aMCI are more likely to develop Alzheimer’s disease (AD) [5], it is also likely that impairment in AM is more common in aMCI rather than the non-amnestic subtype. This aspect can certainly influence the generalizability of the results of this systematic review, but it gives us an overlook of autobiographical memory performance in patients with aMCI.

All the studies included in this systematic review report an impairment in the episodic component of AM in patients with aMCI compared to healthy controls. This finding can have different effects on MCI patients. Indeed, research has demonstrated that people rely on their autobiographical memories to achieve various social, practical, and psychological goals [52]. Indeed, AM has important functions in three main domains: social, directive, and self. The social function involves using AM to connect with others. The directive function refers to using AM for solving problems and planning future behavior, while the self function refers to the fact that people can use AM to develop, maintain, and express an enduring self-concept [52].

Our results demonstrate a more controversial situation concerning the semantic component of AM.

Past research investigating AM in healthy aging has shown that age-related decline is particularly marked in episodic autobiographical memory rather than the semantic component [40]. For what concerns pathological aging, specifically MCI, it has been shown that patients with aMCI typically show a malfunctioning of the hippocampus [17], and this could cause an isolated impairment of episodic memory in patients with MCI [15]. On the other hand, it has been theorized that semantic memory is less dependent on the hippocampus and, therefore, could be less affected by the neuropathology associated with MCI [16].

Our systematic review confirms these hypotheses. Regardless of the utilized test, the episodic component is impaired in all the included studies. Instead, only seven studies [22,23,25,28,30,31,39] have found a worse semantic performance in patients with MCI when compared to the HC group. In addition, three studies [15,16,27] found the opposite effect, i.e., more semantic details in patients’ recollections compared to controls. This result could be explained by the fact that the latter assessed AM following the standardized scoring procedure developed by Levine et al. [40]. This procedure allows researchers to derive both episodic and semantic information using the same test by segmenting a single transcribed autobiographical narrative into internal event-specific and external semantic details. On the other hand, most authors use separate tests to assess the different components of AM. For example, the most widely used measure is the autobiographical memory interview (AMI) [42]. The AMI is a semi-structured interview consisting of two parts; each independently assesses the two AM components, i.e., the episodic and semantic ones [42].

Therefore, the differences in the semantic memory scores could be attributed to these different assessment procedures. Another possible interpretation could be that, in Levine’s procedure, confabulations (which are inaccurate or false narratives produced to give information about the world or the “self”) [53] could be scored as semantic (external) details; therefore, resulting in a better semantic performance for patients with MCI.

Another important aspect to consider is the temporal gradient of autobiographical memories. Indeed, past research has shown evidence of better preservation of AM for older memories than for more recent ones, and this effect is known as Ribot’s law [54].

For what concerns episodic memory, seven studies [24,25,26,28,31,37,39] confirmed Ribot’s law for patients with aMCI. However, three studies [16,34,35] found the opposite effect. The methodological assessment of AM could explain these results. Two out of the three studies that found the opposite effect used Levine’s protocol. It could be possible that by considering the internal/external details, the temporal gradient could be inverted.

On the other hand, for what concerns semantic memory, only three studies [24,25,31] confirmed Ribot’s law for semantic memory, while four articles [16,21,28,39] found the opposite effect. This aspect could mean that semantic memory is more easily accessible for recent experiences than for older ones.

### 4.1. Limits, Implications, and Suggestions for the Future

This review highlighted several limitations in examining autobiographical memory performance in patients with MCI. The main limitation is that MCI is a varied phenomenon. In a recent systematic review [55], authors highlighted the difficulty of diagnosing MCI. They found that MCI prevalence rates range from 1.2 to 87%. This aspect can be attributed to the lack of a comprehensive standardized neuropsychological evaluation to delineate the aging profile associated with MCI. Therefore, studying autobiographical memory in these patients can be affected by the heterogeneity of this nosological category. Moreover, 20 out of 21 studies focused only on 1 MCI subtype, i.e., aMCI. This aspect is another limitation since it prevents further generalizability of the results. Furthermore, considering only individuals classified as aMCI is difficult to understand. In fact, the classification of a person as having aMCI is generally based on poor performance in tests that evaluate verbal short- or long-term memory, using tests such as memory span, Rey’s words, etc., which are all tests that do not assess semantic or episodic aspects of memory. Furthermore, it is well known that people with MCI have dysfunctions in simple [56] and higher-order executive functions [57]. Can it be ruled out that people with executive dysfunction do not have problems with autobiographical memory? Therefore, it cannot be excluded that patients classified as naMCI may present a decline in the episodic and/or semantic components of autobiographical memory. Therefore, the selective choice of subjects classified as aMCI in research on autobiographical memory is based on an unjustified assumption, namely the equivalence of the various forms of memory.

Another limitation could be represented by the partial recovery of articles, including studies published only in some languages that could have excluded relevant information, thus undermining the generalizability of our results.

Regardless of these limitations, at the end of this review emerges an actual need to investigate this specific type of memory in pathological aging. Considering the results of this review, it would be desirable for future research to investigate this aspect to find some gravity indicators of the MCI pathological profile. Moreover, it could be useful to focus on the controversial results related to autobiographical semantic memory, developing a new task more sensitive to detecting changes in this specific component. Finally, it would be interesting to study whether it is possible to draw a relationship between the native language and performance in autobiographical semantic memory.

### 4.2. Conclusions

Overall, this systematic review highlights the presence of impairments in the autobiographical memory performance in patients with MCI. Specifically, all the included studies confirmed that episodic AM is impaired in patients with MCI, while the situation is more controversial when assessing the semantic component.

Thanks to AM, individuals can mentally travel back, acquiring a sense of “self” that can exist in the subjective time [7]. This special form of memory is crucial for developing and maintaining a sense of identity [12]. Moreover, AM has been shown to influence social interactions and abilities such as problem solving and planning [52]. Therefore, starting from the evidence of this systematic work, further studies should detect and deepen the cognitive and emotional mechanisms that undermine AM performance, allowing the development of specific interventions targeting these mechanisms [58]. Indeed, it has been shown that impairments in this memory system are frequent in older adults with depression [59]. For this reason, interventions that alleviate stress and improve mood can enhance AM. Examples are reminiscence therapy and life review, related but distinct interventions that enhance mood and cognitive functions [58]. However, standardized methods of AM stimulation for rehabilitating patients with Alzheimer’s disease or MCI are underdeveloped [60]. An exception is represented by the REMau program (réminiscence autobiographique) [60], whose goal is to improve both the episodic and semantic components of AM, focusing on bettering the orientation in time and the chronology of personal events and teaching strategies for accessing memories of the targeted event. This program showed benefits not only for the AM performance but also for the patients’ moods. Therefore, it is extremely important to develop more standardized methods targeting AM in order to improve the cognitive and psychological conditions of the elderly.

In conclusion, past autobiographical memories are extremely significant to older people, as well as to their caregivers, since they are associated with one’s identity and emotional state, as well as with mood, social functioning, and abilities such as problem solving. This systematic review has highlighted the presence of AM impairment in patients with MCI. These impairments could worsen MCI patients’ performances in many domains, not only related to cognitive functioning, and can be affected by many impaired aspects in elderly people, such as sleep quality and mental health [61,62]. Therefore, it is extremely important to investigate these aspects further, also considering some relevant aspects, such as cognitive reserve [63], in order to develop psychological interventions to improve memory of the past.

## Figures and Tables

**Table 1:** Search syntax.

Database	Keywords	Restrictions	No. of Articles	Duplicates	Total
PubMed	(“mild cognitive impairment” OR MCI) AND (elder* OR aged OR old* OR geriatric OR senior OR aging) AND (“autobiograph* memor*”)	Languages: English, Italian, French, Spanish.	54		
Web of Science	(“mild cognitive impairment” OR MCI) AND (elder* OR aged OR old* OR geriatric OR senior OR aging) AND (“autobiograph* memor*”)	Languages: English, Italian, French, Spanish.	148		
Scopus	(“mild cognitive impairment” OR MCI) AND (elder* OR aged OR old* OR geriatric OR senior OR aging) AND (“autobiograph* memor*”)	Languages: English, Italian, French, Spanish.	2794		
PsycInfo	(“mild cognitive impairment” OR MCI) AND (elder* OR aged OR old* OR geriatric OR senior OR aging) AND (“autobiograph* memor*”)	Languages: English, Italian, French, Spanish.	48		
		TOTAL	3044	222	2822

**Table 2:** Bias assessment.

Study	Attrition Bias	Reporting Bias	Sample Bias	Measurement Bias
Barnabe et al., 2012 [21]	−	−	−	−
Bastin et al., 2013 [34]	−	−	−	+
Berna et al., 2012 [35]	−	−	−	+
Bizzozero et al., 2012 [22]	−	−	−	−
Buckley et al., 2014 [29]	−	−	+	−
Buckley et al., 2014 [30]	−	−	+	−
Davidson et al., 2016 [36]	−	−	−	+
De Simone et al., 2017 [37]	−	−	−	+
Donix et al., 2010 [38]	−	−	−	+
Gamboz et al., 2010 [15]	−	−	+	+
Irish et al., 2010 [23]	−	−	−	−
Leyhe et al., 2009 [24]	−	−	−	−
Meléndez et al., 2016 [31]	−	−	+	−
Meléndez et al., 2019 [32]	−	−	+	+
Meléndez et al., 2021 [33]	−	−	+	−
Müller et al., 2013 [25]	−	−	−	−
Müller et al., 2016 [26]	−	−	−	−
Murphy et al., 2008 [16]	−	−	−	−
Serra et al., 2020 [39]	−	−	−	+
Sheldon et al., 2015 [27]	−	−	−	−
Tramoni et al., 2012 [28]	−	−	−	−

“+” high risk of bias; “−” low risk of bias.

## Data Availability

Not applicable.

## Abbreviations

ADL	Activities of Daily Living
AI	Autobiographical Interview test
aMCI	amnestic mild cognitive impairment
AM/ABM/AuM	autobiographical memory
AMI	Autobiographical Memory Interview
AMT	Autobiographical Memory Test
BNT	Boston Naming Test
BVMT-R	Brief Visuospatial Memory Test-Revised
CDR	Clinical Dementia Rating scale
CDT	Clock Drawing Test
CERAD	Consortium to Establish a Registry for Alzheimer’s Disease test battery
CVLT	California Verbal Learning Test
DS	Digit Span
DSB	Digit Span Backward
DSF	Digit Span Forward
EAMI	Episodic Autobiographical Memory Interview
FAB	Frontal Assessment Battery
FFS	Fruit and Furniture Switching
GDS	Global Deterioration Scale
HAWIE-R	Hamburg-Wechsler-Intelligenztest fur Erwachsene Revision
HC	Healthy Control
HVLT-R	Hopkins Verbal Learning Test–Revised
IADL	Instrumental ADL
LM	Logical Memory
MCI	mild cognitive impairment
MDB	Mental Deterioration Battery
MoCA	Montreal Cognitive Assessment
MODA	Milan Overall Dementia Assessment
NAI	Nurnberger-Alters-Inventar
NART	National Adult Reading Test
n.s.	No significant differences
RCFT	Rey Complex Figure Test
Rey-I	Rey Immediate
Rey-D	Rey Delayed
RL/RI-16	rappel libre/rappel indicé à 16 items
SD	standard deviation
TAVEC-I	Spain–Complutense Verbal Learning Test immediate
TAVEC-D	Spain–Complutense Verbal Learning Test delayed
TBR	Barcelona Test Revised
TMT	Trail Making Test
VFTC	Verbal Fluency Test Categorical
VFTP	Verbal Fluency Test Phonological
WAIS	Wechsler Adult Intelligence Scale
WCST	Wisconsin Card Sorting Test
WMS	Wechsler Memory Scale

## References

[1] Albert M.S., DeKosky S.T., Dickson D., Dubois B., Feldman H.H., Fox N.C., Gamst A., Holtzman D.M., Jagust W.J., Petersen R.C. (2011). The diagnosis of mild cognitive impairment due to Alzheimer’s disease: Recommendations from the National Institute on Aging-Alzheimer’s Association workgroups on diagnostic guidelines for Alzheimer’s disease. Alzheimer’s Dement..

[2] Petersen R.C., Smith G.E., Waring S.C., Ivnik R.J., Tangalos E.G., Kokmen E. (1999). Mild cognitive impairment: Clinical characterization and outcome. Arch. Neurol..

[3] Petersen R.C., Knopman D.S. (2006). MCI is a clinically useful concept. Int. Psychogeriatr..

[4] Petersen R.C. (2011). Clinical practice. Mild cognitive impairment. N. Engl. J. Med..

[5] Petersen R.C., Doody R., Kurz A., Mohs R.C., Morris J.C., Rabins P.V., Ritchie K., Rossor M., Thal L., Winblad B. (2001). Current concepts in mild cognitive impairment. Arch. Neurol..

[6] Welze H., Markowitsch H. (2005). Towards a bio-psycho-social model of autobiographical memory. Memory.

[7] Tulving E. (2002). Episodic memory: From mind to brain. Annu. Rev. Psychol..

[8] Nelson K., Fivush R. (2020). The development of autobiographical memory, autobiographical narratives, and autobiographical consciousness. Psychol. Rep..

[9] Nyberg L. (1996). Classifying human long-term memory: Evidence from converging dissociations. Eur. J. Cogn. Psychol..

[10] Tulving E., Schacter D.L., Mclachlan D.R., Moscovitch M. (1988). Priming of semantic autobiographical knowledge: A case study of retrograde amnesia. Brain Cogn..

[11] Piolino P., Desgranges B., Benali K., Eustache F. (2002). Episodic and semantic remote autobiographical memory in ageing. Memory.

[12] Fivush R. (2011). The development of autobiographical memory. Annu. Rev. Psychol..

[13] El Haj M., Antoine P., Nandrino J.L., Kapogiannis D. (2015). Autobiographical memory decline in Alzheimer’s disease, a theoretical and clinical overview. Ageing Res. Rev..

[14] El Haj M., Gallouj K., Antoine P. (2019). Mental imagery and autobiographical memory in Alzheimer’s disease. Neuropsychology.

[15] Gamboz N., De Vito S., Brandimonte M.A., Pappalardo S., Galeone F., Iavarone A., Della Sala S. (2010). Episodic future thinking in amnesic mild cognitive impairment. Neuropsychologia.

[16] Murphy K.J., Troyer A.K., Levine B., Moscovitch M. (2008). Episodic, but not semantic, autobiographical memory is reduced in amnestic mild cognitive impairment. Neuropsychologia.

[17] Jack C.R., Petersen R.C., Xu Y., O’brien P.C., Smith G.E., Ivnik R.J., Boeve B.F., Tangalos E.G., Kokmen E. (2000). Rates of hippocampal atrophy correlate with change in clinical status in aging and AD. Neurology.

[18] Liberati A., Altman D.G., Tetzlaff J., Mulrow C., Gøtzsche P.C., Ioannidis J.P., Moher D. (2009). The PRISMA statement for reporting systematic reviews and meta-analyses of studies that evaluate health care interventions: Explanation and elaboration. Ann. Intern. Med..

[19] Moher D., Liberati A., Tetzlaff J., Altman D.G., Altman D., Antes G., Tugwell P. (2009). Preferred reporting items for systematic reviews and meta-analyses: The PRISMA statement (Chinese edition). J. Chin. Integr. Med..

[20] Higgins J.P., Altman D.G., Gøtzsche P.C., Jüni P., Moher D., Oxman A.D., Savović J., Schulz K.F., Weeks L., Sterne J.A. (2011). The Cochrane Collaboration’s tool for assessing risk of bias in randomised trials. BMJ.

[21] Barnabe A., Whitehead V., Pilon R., Arsenault-Lapierre G., Chertkow H. (2012). Autobiographical memory in mild cognitive impairment and Alzheimer’s disease: A comparison between the Levine and Kopelman interview methodologies. Hippocampus.

[22] Bizzozero I., Lucchelli F., Saetti M.C., Spinnler H. (2012). Autobiographical memory in amnestic mild cognitive impairment. Neurol. Sci..

[23] Irish M., Lawlor B.A., O’Mara S.M., Coen R.F. (2010). Exploring the recollective experience during autobiographical memory retrieval in amnestic mild cognitive impairment. J. Int. Neuropsychol. Soc..

[24] Leyhe T., Müller S., Milian M., Eschweiler G.W., Saur R. (2009). Impairment of episodic and semantic autobiographical memory in patients with mild cognitive impairment and early Alzheimer’s disease. Neuropsychologia.

[25] Müller S., Saur R., Greve B., Melms A., Hautzinger M., Fallgatter A.J., Leyhe T. (2013). Similar autobiographical memory impairment in long-term secondary progressive multiple sclerosis and Alzheimer’s disease. Mult. Scler. J..

[26] Müller S., Mychajliw C., Reichert C., Melcher T., Leyhe T. (2016). Autobiographical memory performance in Alzheimer’s disease depends on retrieval frequency. J. Alzheimer’s Dis..

[27] Sheldon S., Vandermorris S., Al-Haj M., Cohen S., Winocur G., Moscovitch M. (2015). Ill-defined problem solving in amnestic mild cognitive impairment: Linking episodic memory to effective solution generation. Neuropsychologia.

[28] Tramoni E., Felician O., Koric L., Balzamo M., Joubert S., Ceccaldi M. (2012). Alteration of autobiographical memory in amnestic mild cognitive impairment. Cortex.

[29] Buckley R.F., Saling M.M., Irish M., Ames D., Rowe C.C., Villemagne V.L., Lautenschlager N.T., Maruff P., Macaulay S.L., Martins R.N. (2014). Autobiographical narratives relate to Alzheimer’s disease biomarkers in older adults. Int. Psychogeriatr..

[30] Buckley R.F., Saling M.M., Irish M., Ames D., Rowe C.C., Lautenschlager N.T., Maruff P., Macaulay S.L., Martins R.N., Masters C.L. (2014). Personal memory function in mild cognitive impairment and subjective memory complaints: Results from the Australian Imaging, Biomarkers, and Lifestyle (AIBL) Study of Ageing. J. Alzheimer’s Dis..

[31] Meléndez J.C., Redondo R., Torres M., Mayordomo T., Sales A. (2016). Autobiographical memory for the differential diagnosis of cognitive pathology in aging. Geriatr. Gerontol. Int..

[32] Meléndez J.C., Escudero Torrella J., Satorres Pons E., Pitarque Gracia L.A. (2019). Type of memory and emotional valence in healthy aging, mild cognitive impairment, and Alzheimer’s disease. Psicothema.

[33] Meléndez J.C., Pitarque A., Delhom I., Real E., Abella M., Satorres E. (2021). A Longitudinal Study of Episodic and Semantic Autobiographical Memory in aMCI and Alzheimer’s Disease Patients. Int. J. Environ. Res. Public Health.

[34] Bastin C., Feyers D., Jedidi H., Bahri M.A., Degueldre C., Lemaire C., Collette F., Salmon E. (2013). Episodi. autobiographical memory in amnestic mild cognitive impairment: What are the neural correlates?. Hum. Brain Mapp..

[35] Berna F., Schönknecht P., Seidl U., Toro P., Schröder J. (2012). Episodic autobiographical memory in normal aging and mild cognitive impairment: A population-based study. Psychiatry Res..

[36] Davidson P.S., Cooper L., Taler V. (2016). Remembering a visit to the psychology lab: Implications of Mild Cognitive Impairment. Neuropsychologia.

[37] De Simone M.S., Fadda L., Perri R., De Tollis M., Aloisi M., Caltagirone C., Carlesimo G.A. (2017). Retrograde amnesia for episodic and semantic memories in amnestic mild cognitive impairment. J. Alzheimer’s Dis..

[38] Donix M., Brons C., Jurjanz L., Poettrich K., Winiecki P., Holthoff V.A. (2010). Overgenerality of autobiographical memory in people with amnestic mild cognitive impairment and early Alzheimer’s disease. Arch. Clin. Neuropsychol..

[39] Serra L., Bozzali M., Fadda L., De Simone M.S., Bruschini M., Perri R., Caltagirone C., Carlesimo G.A. (2020). The role of hippocampus in the retrieval of autobiographical memories in patients with amnestic Mild Cognitive Impairment due to Alzheimer’s disease. J. Neuropsychol..

[40] Levine B., Svoboda E., Hay J.F., Winocur G., Moscovitch M. (2002). Aging and autobiographical memory: Dissociating episodic from semantic retrieval. Psychol. Aging.

[41] Moscovitch M., Winocur G. (1992). The neuropsychology of memory and aging. The Handbook of Aging and Cognition.

[42] Kopelman M.D., Wilson B.A., Baddeley A.D. (1989). The autobiographical memory interview: A new assessment of autobiographical and personal semantic memory in amnesic patients. J. Clin. Exp. Neuropsychol..

[43] Petersen R.C., Negash S. (2008). Mild cognitive impairment: An overview. CNS Spectr..

[44] Borrini G., Dall’Ora P., Della Sala S., Marinelli L., Spinnler H. (1989). Autobiographical memory. Sensitivity to age and education of a standardized enquiry. Psychol. Med..

[45] Irish M., Lawlor B.A., O’Mara S.M., Coen R.F. (2008). Assessment of behavioural markers of autonoetic consciousness during episodic autobiographical memory retrieval: A preliminary analysis. Behav. Neurol..

[46] Winblad B., Palmer K., Kivipelto M., Jelic V., Fratiglioni L., Wahlund L.O., Nordberg A., Bäckman L., Albert M., Almkvist O. (2004). Mild cognitive impairment–beyond controversies, towards a consensus: Report of the International Working Group on Mild Cognitive Impairment. J. Intern. Med..

[47] Petersen R.C., Caracciolo B., Brayne C., Gauthier S., Jelic V., Fratiglioni L. (2014). Mild cognitive impairment: A concept in evolution. J. Intern. Med..

[48] Petersen R.C. (2004). Mild cognitive impairment as a diagnostic entity. J. Intern. Med..

[49] Williams J.M., Broadbent K. (1986). Autobiographical memory in suicide attempters. J. Abnorm. Psychol..

[50] Buccione I., Fadda L., Serra L., Caltagirone C., Carlesimo G.A. (2008). Retrograde episodic and semantic memory impairment correlates with side of temporal lobe damage. J. Int. Neuropsychol. Soc..

[51] Benjamin M.J., Cifelli A., Garrard P., Caine D., Jones F.W. (2015). The role of working memory and verbal fluency in autobiographical memory in early Alzheimer’s disease and matched controls. Neuropsychologia.

[52] Kulkofsky S., Koh J.B.K. (2009). Why they reminisce: Caregiver reports of the functions of joint reminiscence in early childhood. Memory.

[53] Berrios G.E. (1998). Confabulations: A conceptual history. J. Hist. Neurosci..

[54] Ribot T. (1881). Les Maladies de la Mémoire.

[55] Casagrande M., Marselli G., Agostini F., Forte G., Favieri F., Guarino A. (2022). The complex burden of determining prevalence rates of mild cognitive impairment (MCI): A systematic review. Front. Psychiatry.

[56] Guarino A., Forte G., Giovannoli J., Casagrande M. (2020). Executive functions in the elderly with mild cognitive impairment: A systematic review on motor and cognitive inhibition, conflict control and cognitive flexibility. Aging Ment. Health.

[57] Corbo I., Casagrande M. (2022). Higher-Level Executive Functions in Healthy Elderly and Mild Cognitive Impairment: A Systematic Review. J. Clin. Med..

[58] Allen A.P., Doyle C., Commins S., Roche R.A. (2018). Autobiographical memory, the ageing brain and mechanisms of psychological interventions. Ageing Res. Rev..

[59] Wilson F.C.L., Gregory J.D. (2018). Overgeneral autobiographical memory and depression in older adults: A systematic review. Aging Ment. Health.

[60] Lalanne J., Gallarda T., Piolino P. (2015). “The Castle of Remembrance”: New insights from a cognitive training programme for autobiographical memory in Alzheimer’s disease. Neuropsychol. Rehabil..

[61] Casagrande M., Forte G., Favieri F., Corbo I. (2022). Sleep Quality and Aging: A Systematic Review on Healthy Older People, Mild Cognitive Impairment and Alzheimer’s Disease. Int. J. Environ. Res. Public Health.

[62] Corbo I., Forte G., Favieri F., Casagrande M. (2023). Poor Sleep Quality in Aging: The Association with Mental Health. Int. J. Environ. Res. Public Health.

[63] Corbo I., Marselli G., Di Ciero V., Casagrande M. (2023). The Protective Role of Cognitive Reserve in Mild Cognitive Impairment: A Systematic Review. J. Clin. Med..

